# Carbon Ion Radiotherapy Evokes a Metabolic Reprogramming and Individualized Response in Prostate Cancer

**DOI:** 10.3389/fpubh.2021.777160

**Published:** 2021-12-07

**Authors:** Renli Ning, Yulei Pei, Ping Li, Wei Hu, Yong Deng, Zhengshan Hong, Yun Sun, Qing Zhang, Xiaomao Guo

**Affiliations:** ^1^Department of Research and Development, Shanghai Proton and Heavy Ion Center, Fudan University Cancer Hospital, Shanghai, China; ^2^Shanghai Key Laboratory of Radiation Oncology (20dz2261000), Shanghai, China; ^3^Shanghai Engineering Research Center of Proton and Heavy lon Radiation Therapy, Shanghai, China; ^4^Department of Radiation Oncology, Shanghai Proton and Heavy Ion Center, Fudan University Cancer Hospital, Shanghai, China; ^5^Department of Radiation Oncology, Shanghai Proton and Heavy Ion Center, Shanghai, China; ^6^Department of Research and Development, Shanghai Proton and Heavy Ion Center, Shanghai, China

**Keywords:** prostate cancer, metabolites, carbon ion radiotherapy, metabolic reprogramming, individualized response, metabolite profiles

## Abstract

**Introduction:** Carbon ion radiotherapy (CIRT) is a novel treatment for prostate cancer (PCa). However, the underlying mechanism for the individualized response to CIRT is still not clear. Metabolic reprogramming is essential for tumor growth and proliferation. Although changes in metabolite profiles have been detected in patients with cancer treated with photon radiotherapy, there is limited data regarding CIRT-induced metabolic changes in PCa. Therefore, the study aimed to investigate the impact of metabolic reprogramming on individualized response to CIRT in patients with PCa.

**Materials and Methods:** Urine samples were collected from pathologically confirmed patients with PCa before and after CIRT. A UPLC-MS/MS system was used for metabolite detection. XCMS online, MetDNA, and MS-DIAL were used for peak detection and identification of metabolites. Statistical analysis and metabolic pathway analysis were performed on MetaboAnalyst.

**Results:** A total of 1,701 metabolites were monitored in this research. Principal component analysis (PCA) revealed a change in the patient's urine metabolite profiles following CIRT. Thirty-five metabolites were significantly altered, with the majority of them being amino acids. The arginine biosynthesis and histidine metabolism pathways were the most significantly altered pathways. Hierarchical cluster analysis (HCA) showed that after CIRT, the patients could be clustered into two groups according to their metabolite profiles. The arginine biosynthesis and phenylalanine, tyrosine, and tryptophan biosynthesis pathways are the most significantly discriminated pathways.

**Conclusion:** Our preliminary findings indicate that metabolic reprogramming and inhibition are important mechanisms involved in response to CIRT in patients with PCa. Therefore, changes in urine metabolites could be used to timely assess the individualized response to CIRT.

## Introduction

Carbon ion radiotherapy (CIRT) is a novel and powerful tool to treat prostate cancer (PCa). Studies have shown an excellent five-year biochemical disease-free survival (bDFS) and low levels of late gastrointestinal and genitourinary toxicities (1, 2). This benefit has been attributed to the physical and biological advantages of CIRT that limits dose to normal tissue while allowing for safe dose escalation. In the past 7 years, our center has treated 162 pathologically confirmed patients with PCa with CIRT, and the three-year bDFS reached 93%. However, these patients with PCa showed an individualized treatment response after CIRT. Yet, the underlying mechanism is still not clear. Moreover, there are still no effective indicators that could be used to timely predict treatment response. Patients might have to wait several months for serum total prostate-specific antigen (PSA) and magnetic resonance imaging (MRI) results after the completion of CIRT, which adversely impacts decision-making. Therefore, there is a need to understand the underlying mechanism involved in response to CIRT in patients with PCa, so as to facilitate the identification of suitable treatment response makers and to evaluate the treatment prognosis.

Metabolic reprogramming is one of the main hallmarks of malignancy, in which tumor cells alter their metabolism, microenvironment, and immune cell function to promote their growth, proliferation, and immune evasion (3). Significant changes in the serum metabolite profiles were detected after photon radiotherapy in different malignancies such as hepatocarcinoma and breast cancer (4, 5). However, it has been shown that carbon ion is associated with a different metabolic response toward photon (6). Meanwhile, the impact of CIRT-induced metabolic changes on PCa treatment response is still not known.

A pilot study conducted in Poland evaluated the free serum and urine amino acid profiles in patients with PCa (7). The results of the study indicate that these metabolite parameters might have great performance for PCa detection. Several metabolites have already shown similar or even better performance for PCa detection when compared with PSA (AUC ranging from 0.53 to 0.83) (8). Therefore, metabolites could potentially be used to evaluate early treatment response following CIRT. Nalbantoglu evaluated the PCa treatment response to radiotherapy and showed that the most significant alterations after photon irradiation were linked with the nitrogen, pyrimidine, purine, porphyrin, alanine, aspartate, glutamate, and glycerophospholipid metabolic pathways (9). Cheema found a correlation between individualized radiation toxicities and metabolite profiles (10). However, these studies were based on photon radiotherapy, highlighting the need to evaluate the impact of CIRT on metabolic reprogramming and individualized treatment response in patients with PCa.

As urine contains over 2,500 metabolites, it can be easily used to evaluate global metabolic changes in patients with cancer (11). Therefore, the study aimed to perform a preliminary investigation to assess the impact of metabolic reprogramming on individualized CIRT response in patients with PCa by measuring variations in urine metabolites following CIRT. We expect this primary investigation of CIRT metabolic reprogramming and the individualized response will further step up the PCa CIRT and will also add value to either CIRT or photon radiotherapy for other malignancies.

## Materials and Methods

### Study Samples and Population

From July 2020 to December 2020, 15 patients with pathologically confirmed prostate adenocarcinoma were enrolled in this study. CIRT was delivered using the Siemens IONTRIS particle therapy device. The clinical target volume (CTV) included prostate with or without proximal seminal vesicles based on different risk group types. The median CIRT dose to the prostate was 60.4 GyE (range 55.2–65.6 GyE) in 12–16 fractions and was prescribed to the 99% isodose line. Risk stratification was based on NCCN guidelines version 2.2020. The demographic and clinical characteristics of enrolled patients are summarized in Table 1.

**Table 1:** Patients' demographic and clinical characteristics.

**Characteristics**	**Statistics**	**No of patients** **(*n* = 15)**	**(%)**
Age (years)	73 (50–82)		
T	T1	1	6.7
	T2	9	60
	T3	4	26.7
	Tx	1	6.7
N	M0	15	100
M	N0	14	93.3
	N1	1	6.7
Gleason score	6	7	46.7
	7	4	26.7
	≥8	4	26.7
Risk group for	Low	2	13.3
Localized PCa	Intermediate	6	40
	High	5	33.3
	Very high	1	6.7

*Data are expressed as numbers (n) and percentages (%) T, tumor; N, lymph node; M, metastasis*.

### Sample Collection and Preparation

The patients' urine samples were collected in the 4 h before receiving the first fraction and 4 h after finishing the last fraction and then stored at 4°C immediately after collection. A 0.22-μm membrane filter was used to remove contaminated bacteria, and 800 μl of chilled methanol/acetonitrile (1:1, v/v) solution was added to 200 μl of the thawed samples. The supernatant was extracted from the centrifuged mixture, transferred into a new Eppendorf tube, and evaporated into a dry solid. The dry supernatant was redissolved into 200 μL chilled acetonitrile/water (1:1, v/v) and transferred into smaller sample vials. Quality control (QC) samples were prepared by mixing equal amounts (50 μL) of each sample.

### High-Throughput UPLC-MS/MS Analysis

High-throughput UPLC-MS/MS (high-performance liquid chromatography, coupled to tandem mass spectrometry) analysis of urine samples was performed on an AB SCIEX ExionLCY system combined with AB SCIEX 500R QTOF. The urinary metabolites were separated in an ACQUITY UPLC BEH Amide 1.7 μm (2.1 × 100 mm) column. Two mobile phases were prepared. Mobile phase A contained water with 10 mM NH4FA and 0.1%FA, and mobile phase B was acetonitrile: water = 95:5 (V/V) with 10 mM NH4FA and 0.1%FA. A 17-min gradient was applied as follows: 2 min, 100% B; 11 min, 45% B; 12 min, 45% B; 12.1 min, 100% B; and 17 min, 100% B. Electrospray ionization mode was performed in the mass spectrometry analysis. Three blanks and six replicates of the QC samples were injected at the beginning of the batch for column conditioning, and the QC sample was analyzed every ten injections. Auto-calibrations were performed every five analyses.

### Data Collection and Metabolite Identification

A UPLC-MS/MS was used to acquire the raw data, and XCMS online was used for peak detection and dataset alignment. MetDNA and MS-DIAL software were used for the identification of metabolites. The acquired peak tables were then uploaded onto the MetaboAnalyst for statistical analysis and metabolic pathway analysis. Concentrations of metabolites were represented by peak area and normalized according to the creatinine levels.

### Data Analysis

MetaboAnalyst 5.0 was used to analyze data. A volcano plot consisting of a combination of fold change (FC) analysis and non-parametric tests was used to identify any statistically significant differences in the metabolites between the pre-CIRT samples and post-CIRT samples. The unsupervised principal component analysis (PCA) was performed to detect the significant separation shift between compared groups. Supervised multivariate analysis and partial least-squares discriminant analysis (PLS-DA) were performed to achieve maximum separation among the groups. The sparse PLS-DA (sPLS-DA) algorithm was used to reduce the number of variables (metabolites) to produce robust and easy-to-interpret models. Hierarchical cluster analysis (HCA) was used to separate the metabolite profiles between compared groups. Boxplots showed the minimum, lower quartile, median, upper quartile, and maximum values of metabolite concentrations. Error bars stood for the minimum values to the maximum.

## Results

### The Impact of CIRT on Urine Metabolite Profiles in Patients With PCa

**Figure 1:**
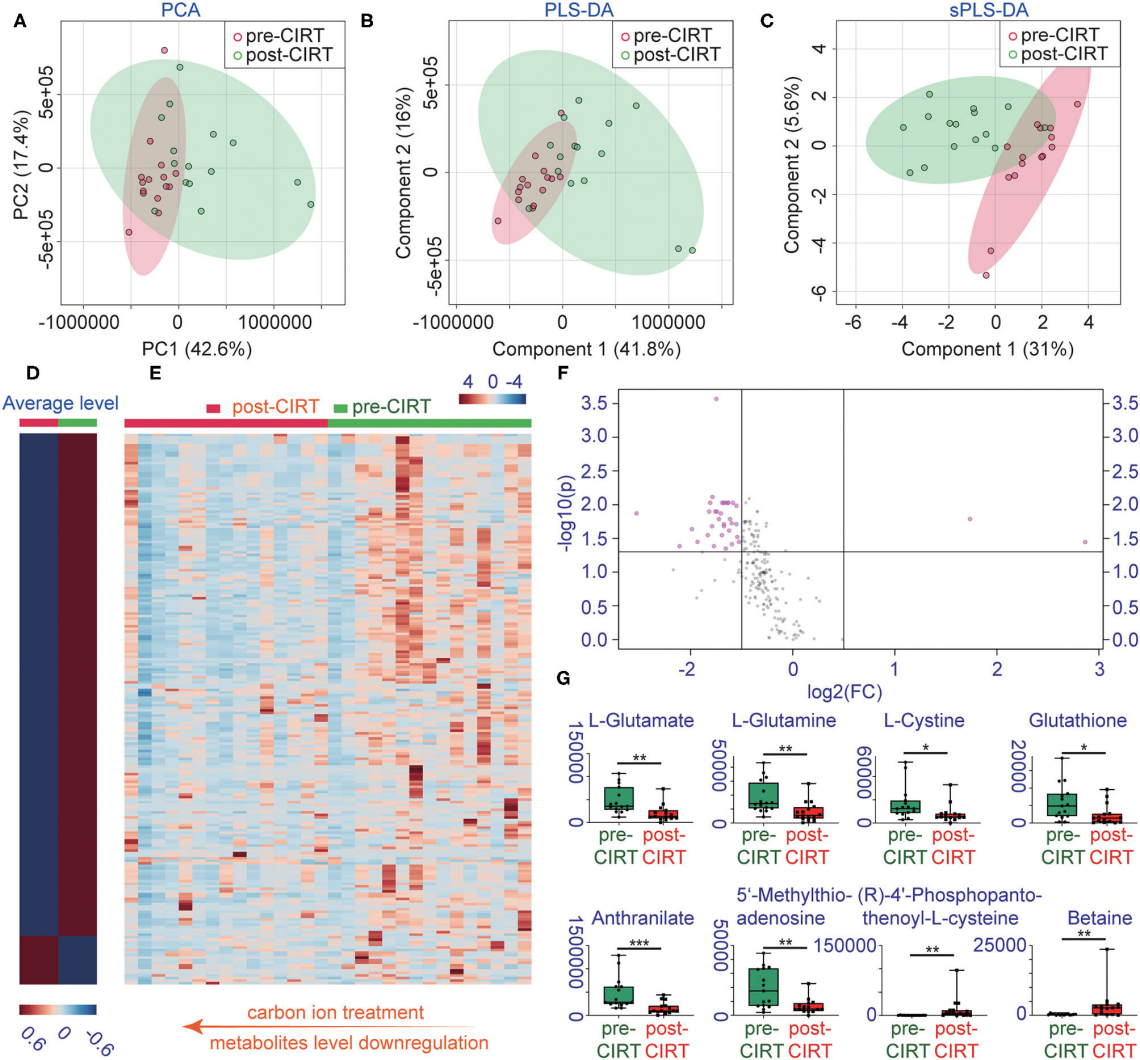
Changes in metabolite profiles before and after CIRT. **(A)** PCA scores, **(B)** partial least squares-discriminant analysis (PLS-DA), **(C)** sparse partial least squares-discriminant analysis (sPLS-DA), **(D)** heat map of the average level of metabolite concentrations in pre-CIRT and post-CIRT urine samples, and **(E)** heat map of the metabolite concentrations in pre-CIRT and post-CIRT urine samples. Upregulated metabolites are shown in red and downregulated in blue. The intensity of the color estimates the magnitude of the change. **(F)** Volcano plot of pre-CIRT samples and post-CIRT samples. Significantly altered metabolites (FDR < 0.05, FC > 2) are indicated in pink; nonsignificantly altered metabolites are indicated in gray. **(G)** Boxplots of L-glutamate, L-glutamine, L-cystine, glutathione, anthranilate, 5'-methylthioadenosine, (R)-4'-phosphopantothenoyl-L-cysteine, betaine.

A total of 1,701 metabolites were monitored by UPLC-MS/MS. Multivariate analysis was performed using PCA, PLS-DA, and sPLS-DA (Figures 1A–C). All samples were analyzed with the unsupervised model PCA to examine possible sample group separations and sample clustering behavior. The PCA score plot revealed significant variations in the patient's urine metabolite profiles before and after CIRT. The pre-CIRT sample clusters are located on the left side of the score plot, and the post-CIRT sample clusters are located on the right side. The small overlap between the two demonstrates the significant difference in the metabolite profiles before and after CIRT. Moreover, the metabolite HCA can clearly discriminate the majority of pre-CIRT samples from the post-CIRT samples (Supplementary Figure 1). The heat map shown in (Figures 1D,E) further confirms that the concentration of metabolites in the urine sample experienced downregulation in most patients after CIRT. The volcano plot identified 35 significantly altered metabolites after CIRT (Figure 1F), with 33 of these metabolites were downregulated after CIRT after CIRT including L-glutamate, L-glutamine, L-cystine, glutathione, anthranilate, 5'-methylthioadenosine, and two were upregulated, including (R)-4'-phosphopantothenoyl-L-cysteine and betaine (Figure 1G). The above results indicate that CIRT can significantly alter the PCa metabolism, mainly by decreasing the amino acid metabolism in urine.

### CIRT-Induced Metabolic Pathway Changes

**Figure 2:**
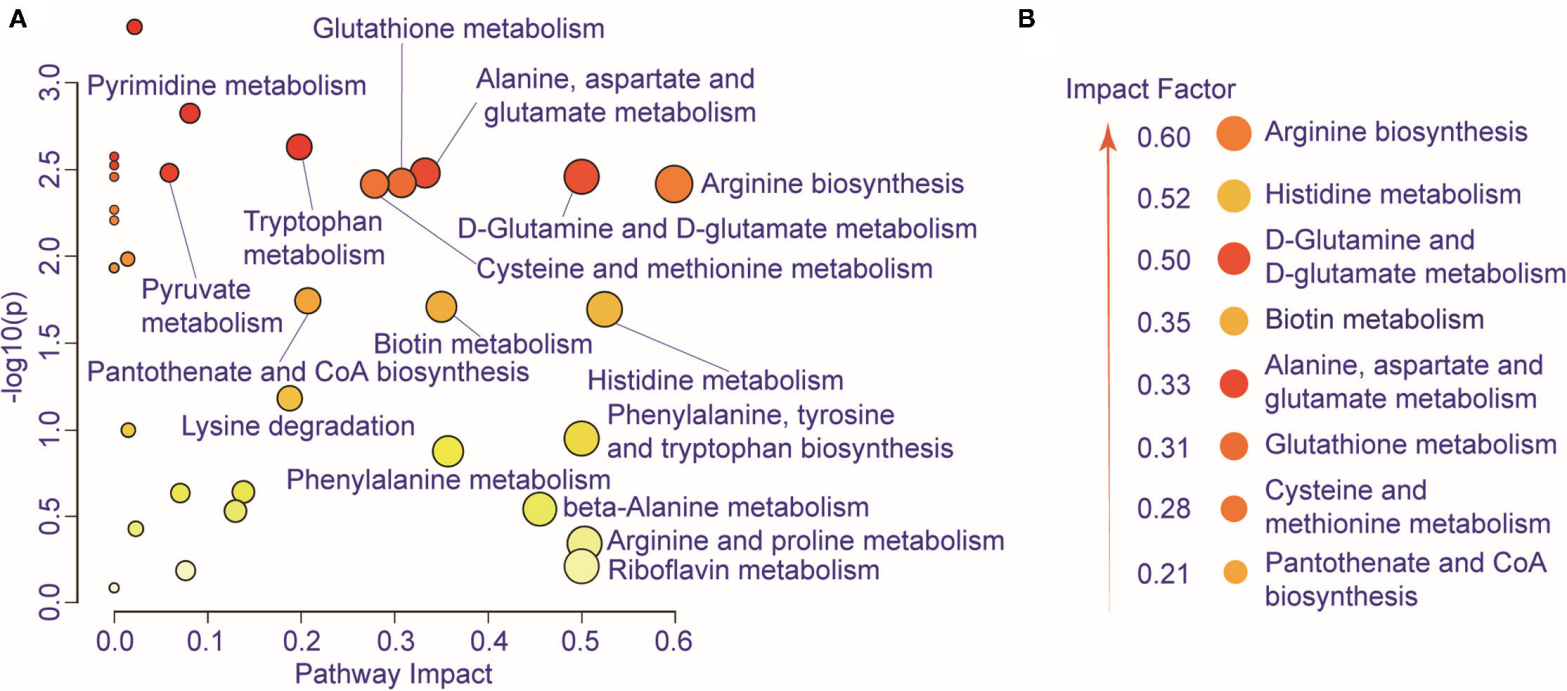
Metabolic pathway alteration by CIRT. **(A)** The bubble chart shows the enrichment of altered metabolite pathways between pre-CIRT samples and post-CIRT samples. The size and color of the bubbles represent the impact and –log10(p) values for each pathway. **(B)** Scheme illustrating the altered pathway sorted by impact factor from top to bottom.

**Figure 3:**
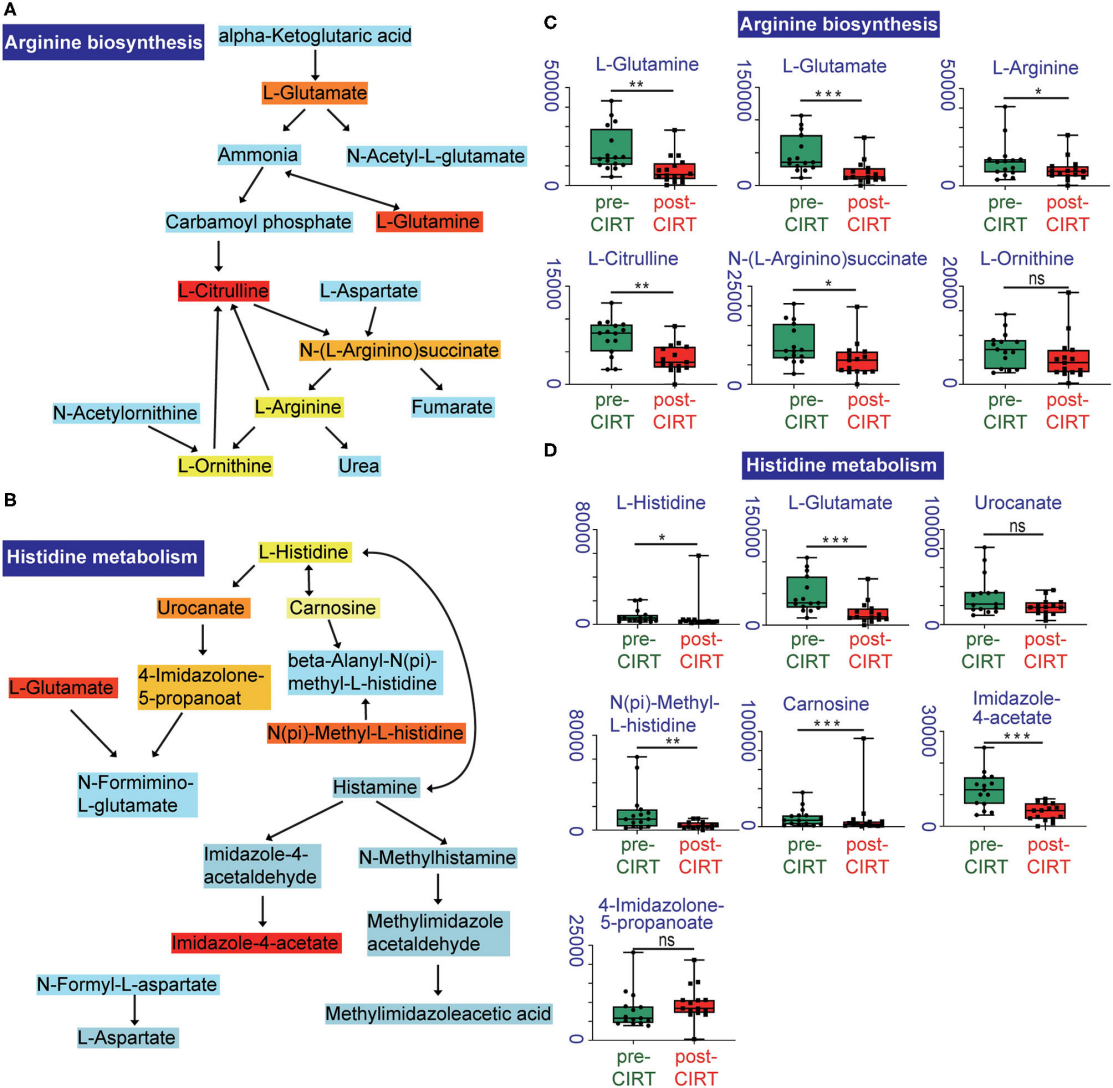
Altered metabolites in arginine biosynthesis and histidine metabolism pathways. Identified compounds within the pathway of arginine biosynthesis **(A)** and histidine metabolism **(B)**. Light blue means that those metabolites are not in our data and are used as background for the enrichment analysis; other colors (varying from yellow to red) indicate the level of significance of the metabolites in the data. Boxplots of L-glutamine, L-glutamate, L-arginine, L-citrulline, N-(L-arginino) succinate, L-ornithine in arginine biosynthesis **(C)**, and L-histidine, L-glutamate, urocanate, N(pi)-methyl-L-histidine, carnosine, imidazole-4-acetate, 4-imidazolone-5-propanoate in histidine metabolism **(D)**. *p < 0.05, **p < 0.01, and ***p < 0.001.

We further performed pathway enrichment analysis of the identified metabolites, and we found that these metabolites could be enriched in eight pathways (FDR < 0.05, impact > 2), including histidine metabolism, arginine biosynthesis, glutathione metabolism, cysteine and methionine metabolism, pantothenate, and CoA biosynthesis, biotin metabolism, alanine, aspartate and glutamate metabolism, D-glutamine and D-glutamate metabolism. These metabolic pathways are part of amino acid metabolism, carbohydrate metabolism, and also vitamins and cofactors metabolism. The bubble plot shown in Figure 2A demonstrates the significance and the impact of each pathway. Figure 2B demonstrates the altered pathway sorted by impact factor from top to bottom. Figures 3A,B shows the metabolites in arginine biosynthesis and histidine metabolism. Supplementary Figures 2, 3 demonstrate the details of the other six pathways that were significantly altered. Supplementary Table 1 shows the FDR and the impact of the enriched metabolite pathways before and after CIRT. The alterations of the arginine biosynthesis and histidine metabolism pathways by CIRT are the most significant. L-glutamine, L-glutamate, L-arginine, L-citrulline, N-(L-arginino) succinate, and L-ornithine in arginine biosynthesis are all downregulated (Figure 3C), and L-histidine, L-glutamate, urocanate, N(pi)-methyl-L-histidine, carnosine, and imidazole-4-acetate in the histidine metabolism are downregulated as well (Figure 3D).

### Potential Metabolite Profile Response Indicators for CIRT

The relation of metabolic clustering with different risk classifications was further explored. Patient risk stratification was performed under the NCCN guidelines. The low-risk and medium-risk patients were considered as a relatively low-risk group, and the high-risk and very high-risk patients were considered as a relatively high-risk group (Supplementary Table 2).

**Figure 4:**
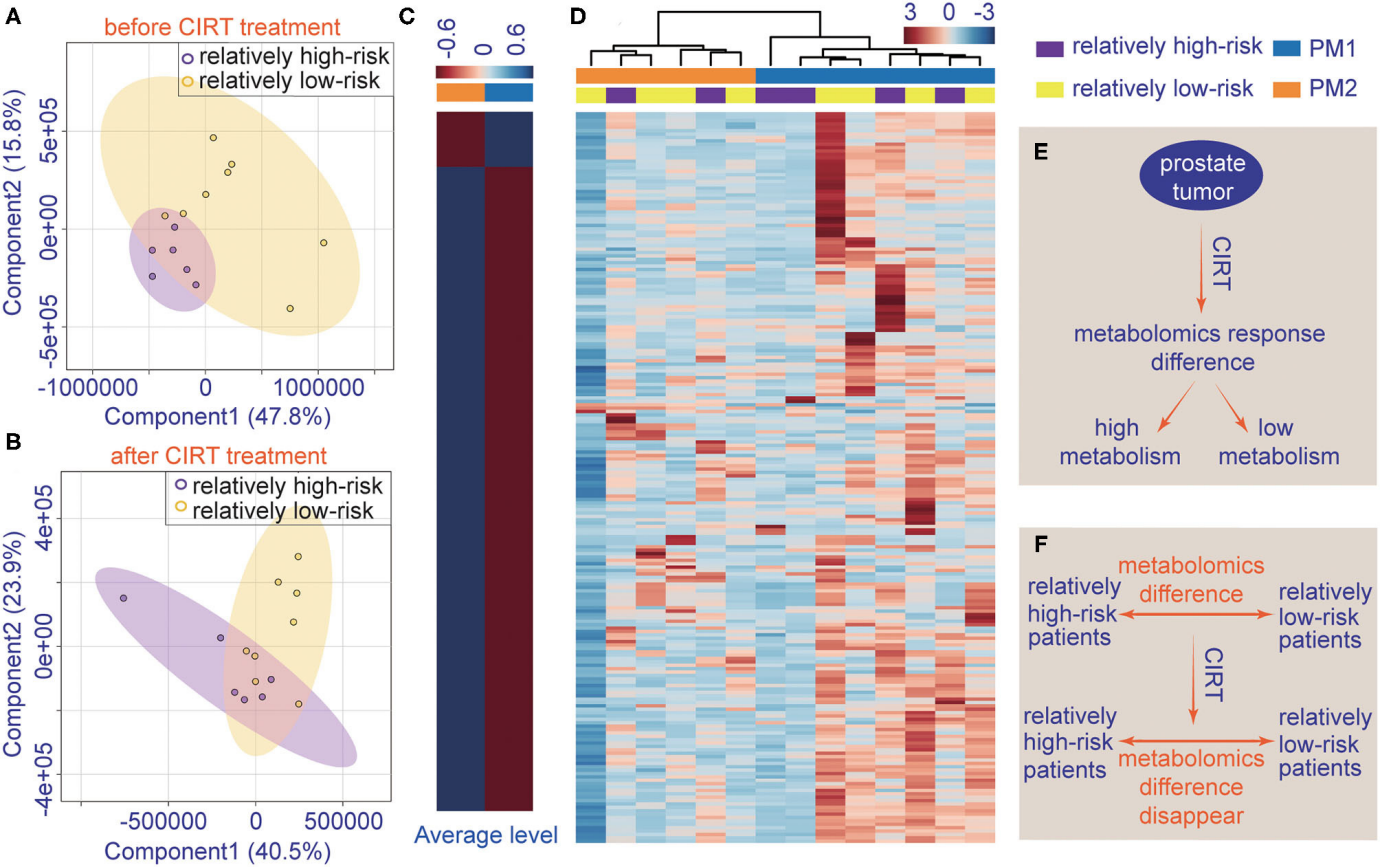
The individualized difference in metabolite profiles among patients after CIRT. **(A)** PLS-DA analysis of relatively low-risk group and the relatively high-risk group shows less overlap in the pre-CIRT samples; **(B)** PLS-DA analysis of relatively low-risk group and the relatively high-risk group shows less overlap in post-CIRT samples; **(C)** heat map of the average level of metabolite concentrations in PM1 and PM2 urine samples. **(D)** HCA of metabolites in post-CIRT samples. Upregulated metabolites are shown in red, and the downregulated metabolites are shown in blue. The intensity of the color indicates the magnitude of the change post-CIRT, **(E)** schematic description of the individualized metabolomic response difference, **(F)** schematic description of CIRT-induced disappearance of metabolomic difference between relatively high-risk and low-risk patients.

The patient's urine metabolites in pre-CIRT were further analyzed by PLS-DA and were clustered into two groups. The results were matched with the risk subtype (Figure 4A). However, the PLS-DA analysis of post-CIRT urine metabolites shows more overlap (Figure 4B), indicating that patients assessed as the same risk subtype no longer represented similar urine metabolite profiles, which means CIRT could significantly decrease the discrimination of the risk stratification.

The HCA also revealed that after CIRT, the patients could be clustered into two groups, PM1 and PM2, according to their metabolic profiles (Figure 4D). This clustering was different from the risk subtype. The PCa patients in the PM2 group had a higher level of urine metabolite concentrations than PM1 group (Figure 4C), which means the patients in the two groups may have different responses to CIRT. Figures 4E,F illustrate the schematic diagrams of this process.

### Metabolic Pathways Enrichment Analysis After CIRT

**Figure 5:**
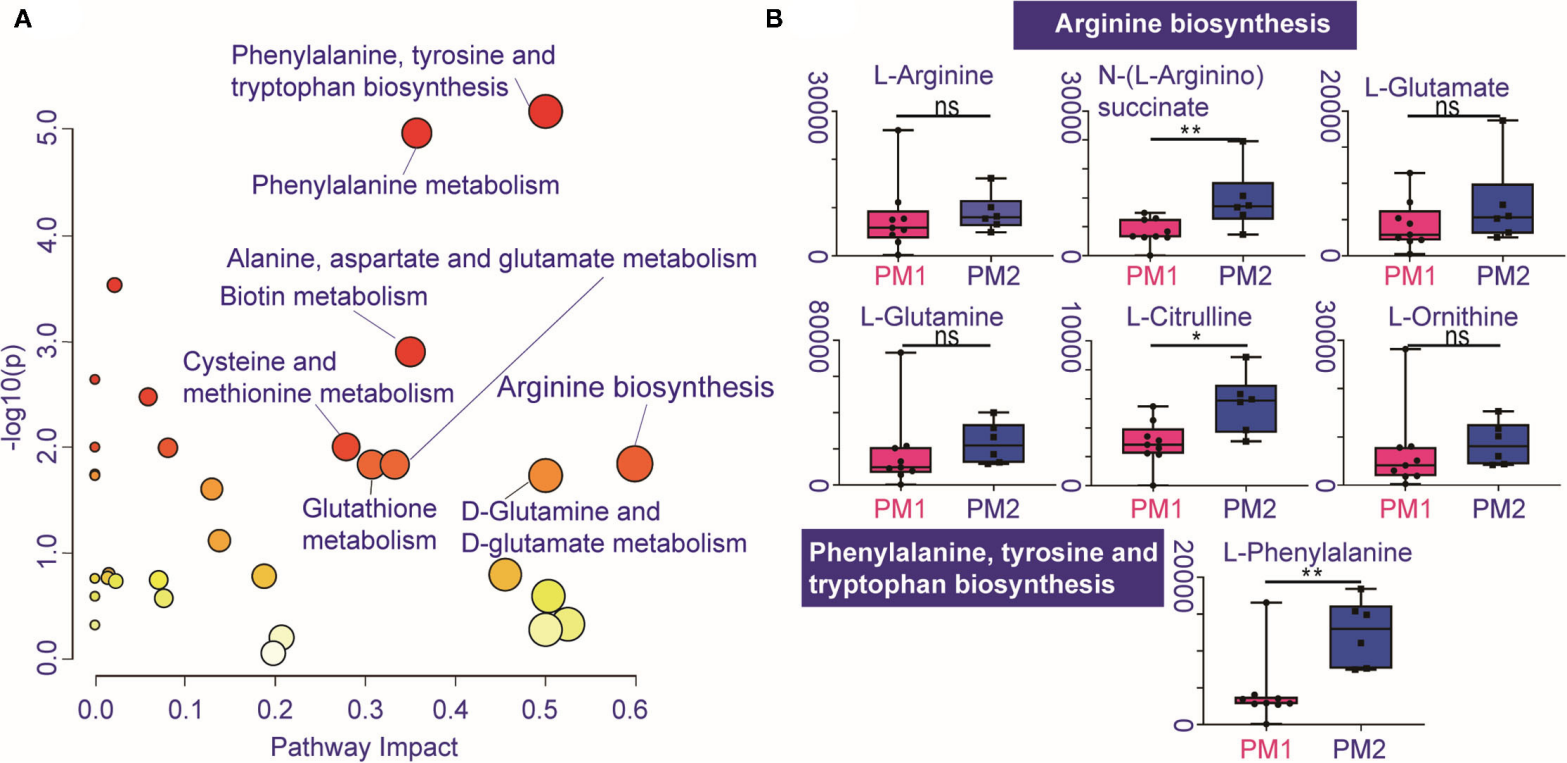
Individualized difference in response to CIRT within the metabolic pathways. **(A)** The bubble chart shows the enrichment pathways of the discriminatory metabolites between PM1 and PM2. The size and color of the bubble represent the impact and –log10(p) values for each pathway, **(B)** boxplots of L-glutamine, L-glutamate, L-arginine, L-citrulline, N-(L-arginino)succinate, L-ornithine in arginine biosynthesis, and L-phenylalanine in phenylalanine, tyrosine, and tryptophan biosynthesis.

Pathway enrichment shows the response diversity of PCa to CIRT. The bubble chart of the discriminatory metabolic pathway is shown in Figure 5A. Supplementary Table 3 demonstrates the FDR and the impact of the enriched pathways in discriminating between metabolites in post-CIRT urine samples. Discriminatory metabolites are mainly enriched in eight pathways (FDR <0.05, impact > 2), including phenylalanine, tyrosine, and tryptophan biosynthesis, phenylalanine metabolism, biotin metabolism, cysteine and methionine metabolism, glutathione metabolism, arginine biosynthesis, alanine, aspartate, and glutamate metabolism, D-glutamine and D-glutamate metabolism. The arginine biosynthesis and the phenylalanine, tyrosine, and tryptophan biosynthesis pathways are the most significant. Figure 5B shows that the concentration of L-glutamine, L-glutamate, L-arginine, L-citrulline, N-(L-arginino) succinate, L-ornithine in the arginine biosynthesis, and L-phenylalanine in phenylalanine, tyrosine, and tryptophan biosynthesis are higher in PM2 when compared with PM1.

## Discussion

Carbon ion radiotherapy is a relatively new radiotherapy technique, and few studies have evaluated the impact of CIRT on cancer metabolism, especially PCa. Therefore, in this study, we evaluated the impact of this new technology on PCa metabolism.

Carbon ion radiotherapy showed a strong ability to inhibit metabolism in PCa. Compared to the concentrations of the metabolites in untreated patients' urine samples, almost all discriminated metabolites (33/35) were downregulated after CIRT. This result demonstrates the ability of CIRT to inhibit tumor metabolism. Moreover, CIRT could generally inhibit most of the metabolic processes involved in the proliferation, metastasis, and finally the progression of PCa. The results in this study primarily suggest that CIRT has the ability to significantly downregulate metabolism in patients with PCa.

Carbon ion radiotherapy inhibited the production of metabolites that are mainly enriched in the arginine biosynthesis and histidine metabolism pathways. The main alteration of amino acid in the process of prostate tumorigenicity involves histidine and arginine metabolism, and also the metabolism of alanine, aspartate, and glutamate, and some aromatic amino acid metabolism (12). The arginine biosynthesis pathway plays a key role, and it is known to be upregulated in PCa progression (13, 14). The deprivation of arginine in cancer cells can lead to dysfunction of mitochondria, reprogramming of transcription, and result in cell death (15). Arginine deprivation therapy for PCa has been found to be an effective treatment (16) and has a strong radiosensitizing impact (17). Moreover, increased metabolism of L-arginine by myeloid cells can result in an impaired lymphocyte response to antigens and tumor growth (18). Therefore, the downregulation of arginine metabolism will inhibit PCa progression and also has the potential to promote antitumor immune effects. Histidine metabolism is another pathway that is significantly inhibited after CIRT. Histidine metabolism has been found elevated in men with T2 PCa, and its metabolite 4-imidazoleacetate shows cumulative effect in serum from T2 to T4 PCa (19). Herein, through a high-resolution metabolomic technique based on UPLC-MS/MS, we found carbon ion downregulated almost all of the amino acid metabolism, especially the histidine, arginine, and glutamine, presenting the unique inhibition effects of the carbon ion beam on PCa. This effect was significantly different from the reported photon radiation effects (20). Considering the role of these amino acids in PCa progression, further exploring the effects of the carbon ion beam on PCa metabolism was necessary for the future.

Interestingly, we found the urine metabolites of these patients with PCa have different responses to CIRT. All patients could be clustered into two groups, PM1 and PM2. PM2 showed relatively higher concentrations of metabolites after CIRT. The clustered result was different from the clinical risk stratification. Therefore, the difference in the concentrations of metabolites between PM1 and PM2 can be attributed to tumor sensitivity to CIRT. PCa in the PM2 group patients seemed to be less sensitive to CIRT when compared with the PCa in the PM1 group. However, long-term follow-up is necessary to confirm the role of urine metabolites as treatment response markers for CIRT in PCa.

The metabolic results also confirm the response to CIRT of PCa in the PM2 group, shown by the higher levels of metabolites related to arginine biosynthesis and also phenylalanine, tyrosine, and tryptophan biosynthesis. These results further confirm that arginine biosynthesis is important for PCa and may play a central role in response to CIRT.

## Conclusion

In this study, CIRT showed its strong ability to inhibit metabolism pathways in PCa. CIRT-induced changes in the metabolite profiles mainly enriched in arginine biosynthesis and histidine metabolism. Urine metabolites of patients with PCa had different responses to CIRT. More sensitive PCa showed lower levels of metabolites in urine samples, especially the arginine biosynthesis and also phenylalanine, tyrosine, and tryptophan biosynthesis pathway. CIRT-evoked metabolic reprogramming seems to be one of the most important underlying mechanisms of CIRT to inhibit PCa. Our preliminary results indicate that some urine metabolites could potentially be used to identify the individualized response to CIRT in patients with PCa. However, further longitudinal studies with a larger sample size are recommended to confirm these results.

## Data Availability Statement

The raw data supporting the conclusions of this article will be made available by the authors, without undue reservation.

## Ethics Statement

The studies involving human participants were reviewed and approved by Shanghai Proton and Heavy Ion Center Institutional Review Board. The patients/participants provided their written informed consent to participate in this study.

## Author Contributions

YP, QZ, and RN finished study design. QZ and YP finished experimental studies. YP and YS finished data analysis. QZ, PL, ZH, YP, WH, and YD collected and proceeded patients' samples and clinical information. YS, QZ, and YP finished manuscript editing. QZ, XG, and YS supervised the study. All authors read and approved the final manuscript.

## Funding

This article was supported by the Science and Technology Development Fund of Shanghai Pudong New Area (PKJ2019-Y07, PKJ2020-Y52, PKJ2019-Y06), the Natural Science Foundation of Shanghai (21ZR1481800).

## Conflict of Interest

The authors declare that the research was conducted in the absence of any commercial or financial relationships that could be construed as a potential conflict of interest.

## Publisher's Note

All claims expressed in this article are solely those of the authors and do not necessarily represent those of their affiliated organizations, or those of the publisher, the editors and the reviewers. Any product that may be evaluated in this article, or claim that may be made by its manufacturer, is not guaranteed or endorsed by the publisher.

## Abbreviations

PCa: Prostate cancer
CIRT: carbon ion radiotherapy
bDFS: biochemical disease-free survival
UPLC-MS/MS: high-performance liquid chromatography, coupled to tandem mass spectrometry
CTV: clinical target volume
T: tumor
N: lymph node
M: metastasis
QC: quality control
ESI: electrospray ionization
MetDNA: Metabolite identification and Dysregulated Network Analysis (http://metdna.zhulab.cn/)
MS-DIAL software: http://prime.psc.riken.jp/
FC: fold change
FDR: false discovery rates
PCA: principal component analysis
PLS-DA: partial least-squares discriminant analysis
sPLS-DA: sparse partial least-squares discriminant analysis
HCA: hierarchical cluster analysis.
