# Supplementary material for: Carbon Ion Radiotherapy Evokes a Metabolic Reprogramming and Individualized Response in Prostate Cancer
Source: Front Public Health. 2021 Dec 7;9:777160. doi: 10.3389/fpubh.2021.777160 (PMC8688694; doi:10.3389/fpubh.2021.777160)
Supplement: Supplementary file 1 [file Data_Sheet_1.DOCX]

**SUPPLEMENTARY FIGURES**


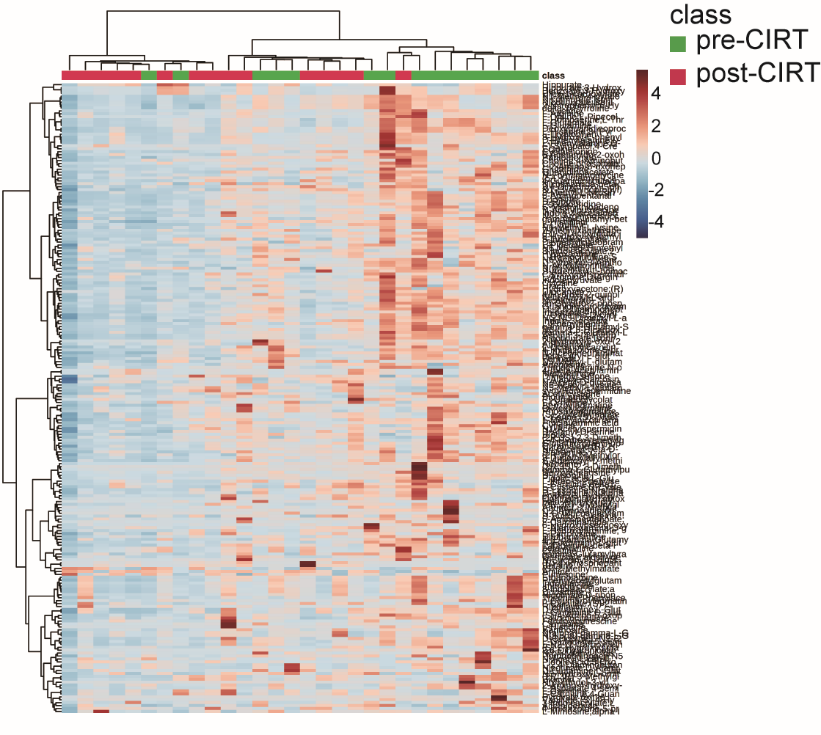


**Supplementary Figure 1**

Hierarchical clustering analysis of all samples. Pre-CIRT is green (n=15) and post-CIRT is red (n=15)


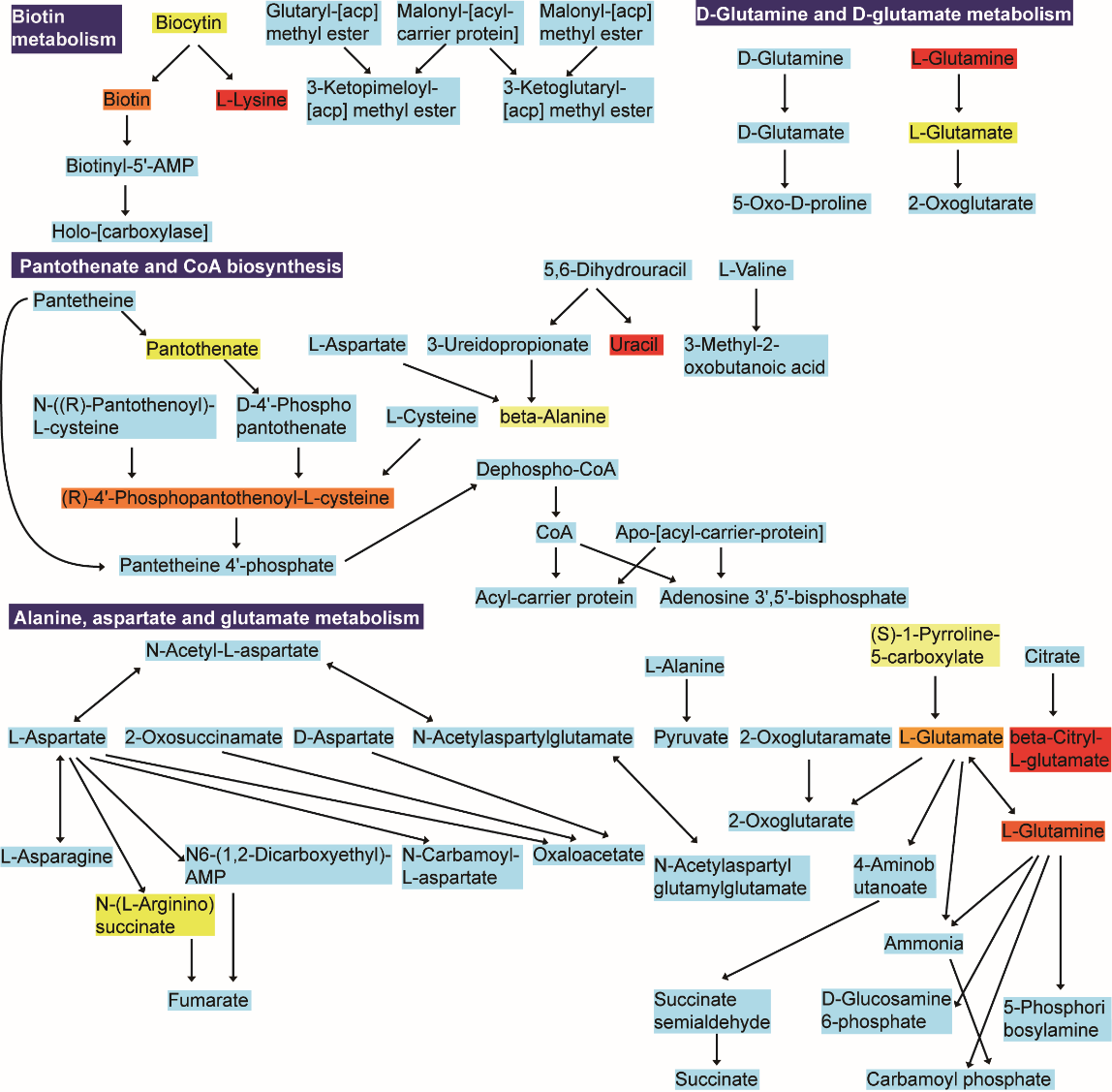


**Supplementary Figure 2**

Identified compound within the pathway of biotin metabolism, D-Glutamine and D-glutamate metabolism, pantothenate and CoA biosynthesis, alanine, aspartate and glutamate metabolism. Light blue means those metabolites are not in our data and are used as background for enrichment analysis; other colors (varying from yellow to red) means the metabolites are in the data with different levels of significance.


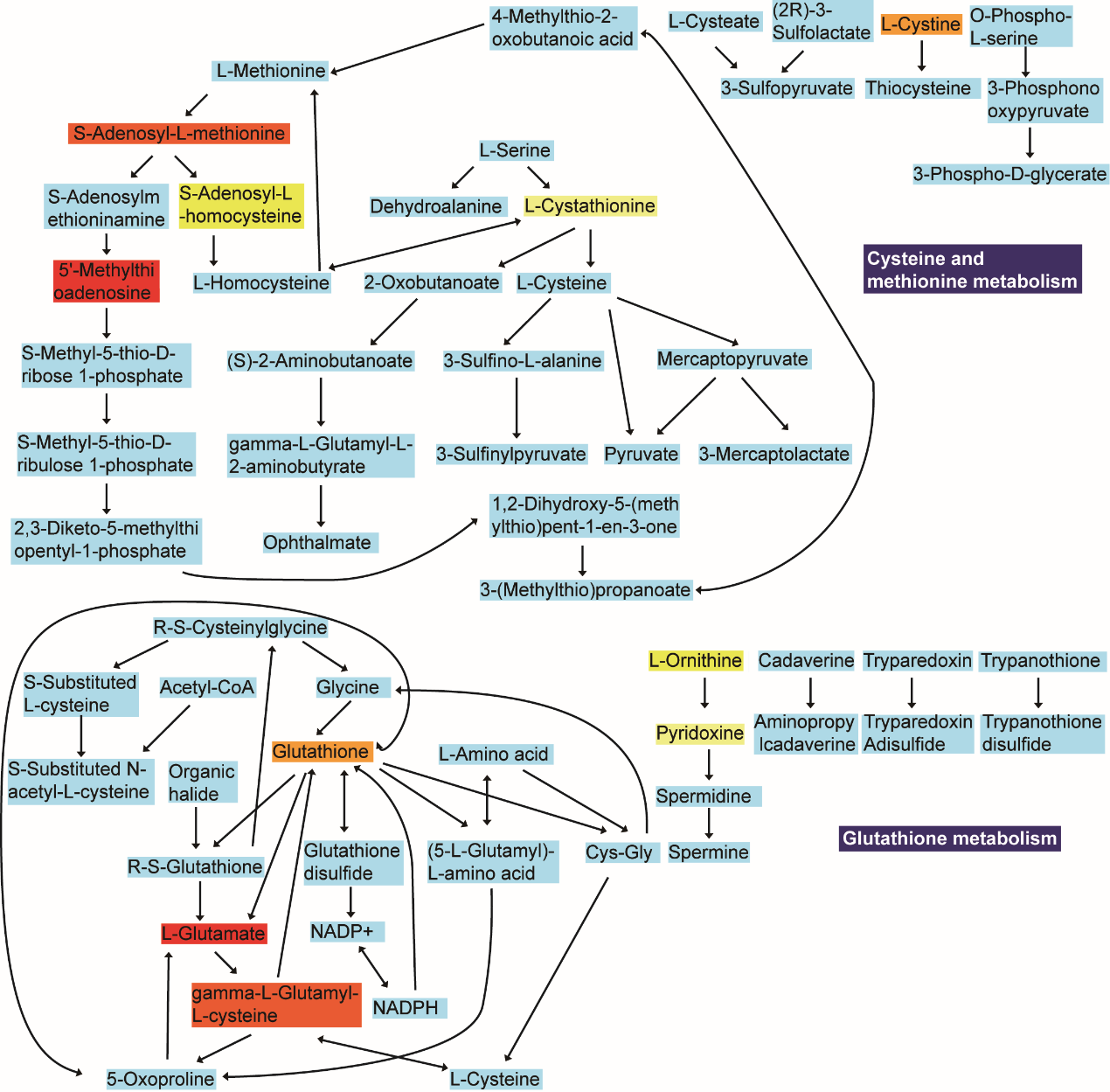


**Supplementary Figure 3**

Identified compound within the pathway of glutathione metabolism, cysteine and methionine metabolism. Light blue means those metabolites are not in our data and are used as background for enrichment analysis; other colors (varying from yellow to red) means the metabolites are in the data with different levels of significance.

**Supplementary Table 1. The FDR and impact of enriched pathways of altered metabolites between pre-CIRT samples and post-CIRT samples.**

| Pathway Name | Match Status | P | FDR | Impact |
| --- | --- | --- | --- | --- |
| Alanine, aspartate and glutamate metabolism | 5/28 | 0.003315 | 0.009313 | 0.33253 |
| D-Glutamine and D-glutamate metabolism | 2/6 | 0.003481 | 0.009313 | 0.5 |
| Glutathione metabolism | 5/28 | 0.003779 | 0.009313 | 0.30741 |
| Cysteine and methionine metabolism | 5/33 | 0.003834 | 0.009313 | 0.27873 |
| Arginine biosynthesis | 6/14 | 0.003835 | 0.009313 | 0.59898 |
| Pantothenate and CoA biosynthesis | 4/19 | 0.01807 | 0.032335 | 0.20714 |
| Biotin metabolism | 3/10 | 0.019576 | 0.032757 | 0.35 |
| Histidine metabolism | 7/16 | 0.020232 | 0.032757 | 0.52458 |

**Supplementary Table 2. Risk subtype of PCa patients.**

| risk group | n | risk subtype | n |
| --- | --- | --- | --- |
| Low | 2 | relatively low-risk | 8 |
| Intermediate | 6 |  |  |
| High | 5 | relatively high-risk | 6 |
| Very high | 1 |  |  |

**Supplementary Table 3. The FDR and impact of enriched pathways of different metabolites between PM1 group and PM2 group**

| Pathway Name | Match Status | P | FDR | Impact |
| --- | --- | --- | --- | --- |
| Phenylalanine, tyrosine and tryptophan biosynthesis | 1/4 | 1.07E-05 | 2.67E-04 | 0.5 |
| Phenylalanine metabolism | 2/10 | 1.57E-05 | 2.67E-04 | 0.35714 |
| Biotin metabolism | 3/10 | 5.86E-04 | 0.004984 | 0.35 |
| Cysteine and methionine metabolism | 5/33 | 0.00615 | 0.026065 | 0.27873 |
| Glutathione metabolism | 5/28 | 0.009347 | 0.026065 | 0.30741 |
| Arginine biosynthesis | 6/14 | 0.009608 | 0.026065 | 0.59898 |
| Alanine, aspartate and glutamate metabolism | 5/28 | 0.009912 | 0.026065 | 0.33253 |
| D-Glutamine and D-glutamate metabolism | 2/6 | 0.013032 | 0.026065 | 0.5 |
